# Supplementary material for: Two-Dimensional Hetero- to Homochiral Phase Transition from Dynamic Adsorption of Barbituric Acid Derivatives
Source: Nanomaterials (Basel). 2023 Aug 10;13(16):2304. doi: 10.3390/nano13162304 (PMC10458813; doi:10.3390/nano13162304)
Supplement: Supplementary file 1 [file nanomaterials-13-02304-s001.zip › nanomaterials-2540889-supplementary.pdf]

Supporting Information

2D Hetero- to Homo- Chiral Phase Transition from Dynamic Adsorption of Barbituric Acid Derivatives

*Fabien Silly [1], Changzhi Dong [2], François Maurel [2] and Xiaonan Sun[2]**

[1] Université Paris-Saclay, CEA, CNRS, SPEC, TITANS F-91191 Gif sur Yvette, France, France;

[2] Université Paris Cité, ITODYS, CNRS UMR 7086, 15 rue Jean Antoine de Baïf, 75013 Paris, France;

AUTHOR INFORMATION

Corresponding Author

sun.xiaonan@u-paris.fr

.

1. Synthesis Chemistry

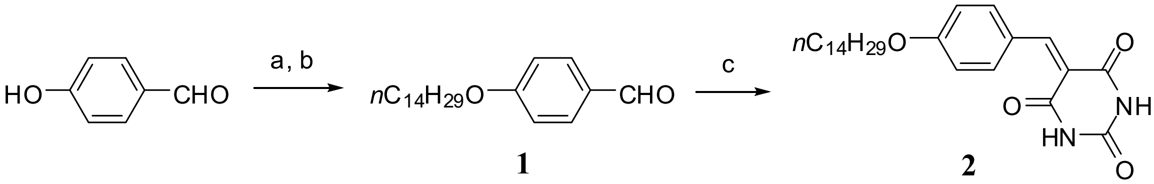

Scheme: Reagents and conditions: (a) NaH, in dry DMF, 0°C; (b) $nC_{14}H_{29}Br$; (c) Barburic acid, cat. piperidinium benzoate, toluene, reflux

Experimental section

All chemicals were of reagent quality and were used without further purification. The purity of each compound was checked by thin-layer chromatography on TLC plastic sheets (silica gel 60F 254, layer thickness 0.2 mm) from Merck. Column chromatography purification was carried out on silica gel 60 (particle size 0.063–0.200 mm) from Merck without any special treatment. All melting points were determined in a digital melting point apparatus (Electrothermal) and are uncorrected. The structures of all compounds were confirmed by IR and 1H NMR spectra. IR spectra were obtained with an ATI Mattson GENESIS SERIES FTIR infrared spectrometer, and 1H NMR spectra were recorded in $CDCl_3$ or DMSO-d6 on a Bruker AC 200 spectrometer using hexamethyldisiloxane (HMDS) as an internal standard. Chemical shifts are reported in parts per million (d) and coupling constants expressed in Hz. Elemental analyses were carried out for C, H and N by the Service Régional de Microanalyse de l'Université Paris Cité and are within ± 0.4% of theoretical values.

4-Tetradecyloxybenzaldehyde (1)

To a suspension of NaH (60% in mineral oil, 25.0 g, 0.625 mol) in DMF (500 mL) at 0 °C was added dropwise a solution of 4-hydroxybenzaldehyde (75.0 g, 0.614 mol) in DMF (100 mL). When no H2 escaped any more from the suspension (about 15 min after addition of NaH), 1-bromotetradecane (172 mL, 0.577 mol) was introduced dropwise and the reaction mixture was stirred at room temperature overnight. The salt NaBr was then filtered and the solvent removed under reduced pressure. The residue was partitioned in ether and water, and the organic layer was washed with water, brine, and then dried over MgSO4. The title compound 1 was obtained as colourless oil (187 g, 95% yield) and used without further purification: 1H NMR (CDCl3) 0.81 (t, 3H, J = 6.1, CH3), 1.19 (m, 22H, (CH2)ch), 1.71 (dt, 2H, J = 6.7, OCH2CH2), 3.97 (t, 2H, J = 6.5, OCH2), 6.90 (d, 2H, J = 8.7, Har), 7.74 (d, 2H, J = 8.7, Har), 9.60 (s, 1H, CHO).

5-(4-Tetradecyloxybenzylidene)pyrimidine-2,4,6-trione (2)

In a flask equipped with a Dean and Stark apparatus were mixed 1 (2.00 g, 6.28 mmol), barbituric acid (0.80 g, 6.28 mmol) and piperidinium benzoate (25 mg, 0.13 mmol) in toluene (40 mL). The mixture was stirred and heated to reflux for 2 h. The clear yellow

solution was cooled slowly to room temperature and the crystals were collected by filtration. A re-crystallization in ethanol provided pure 2 as yellow crystals (1,72 g, 64% yield): mp 219.0-220.5°C; 1H NMR (DMSO-d6, 60°C) 0.84 (t, 3H, J = 6.5, CH_3), 1.23 (m, 22H, (CH_2)ch), 1.71 (dt, 2H, J = 6.1, OCH_2CH_2), 4.08 (t, 2H, J = 6.3, OCH_2), 7.01 (d, 2H, J = 8.8, Har), 7.25 (s, 1H, CH), 8.33 (d, 2H, J = 8.8, Har), 11.00 (s, 1H, NH), 11.13 (s, 1H, NH). Anal. ($C_{25}H_{36}N_2O_4$) C, H, N.

2. Chirality definition

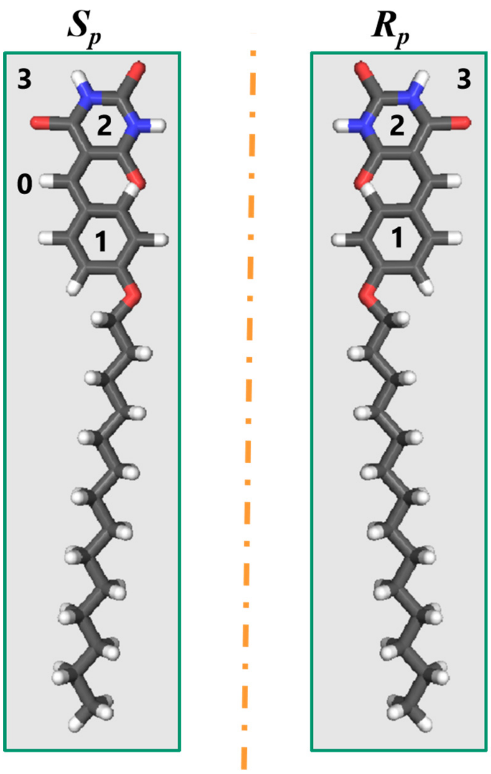

Scheme S1. The surface is treated here as a substituent and is considered to have the highest priority (3). The hydrogen of the CH bridging the two rings has the lowest priority (0). The barbituric ring has priority (2) and the phenyl ring (1). The left configuration can then be defined as S_p and right R_p.

3. STM results

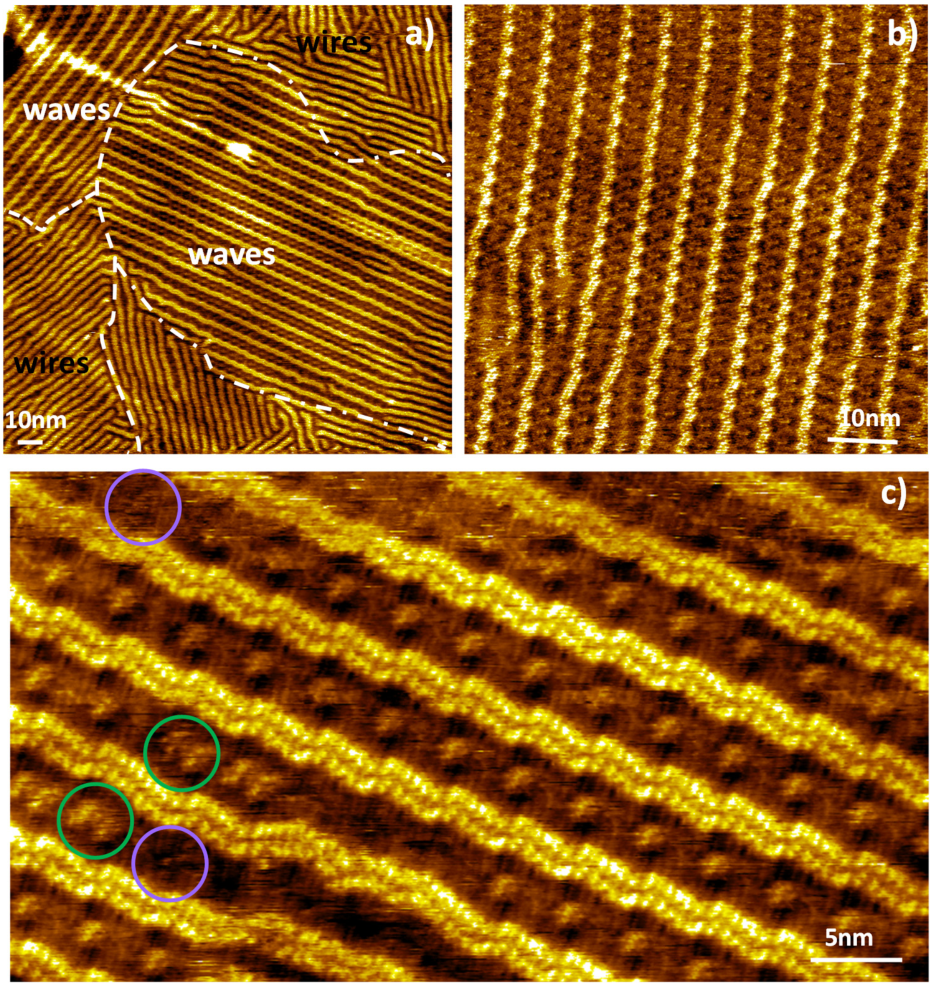

Figure S1. STM images of (a) TDPT "nanowaves" and "nanowires" coexist. 180×180 nm^2, (b) TDPT "nanowaves" 68×66 nm^2, (c) TDPT "nanowaves" host-guest nanoarchitecture, 50×27 nm^2 with four guests, two guest or no guest molecules. I_t = 20-60 pA, U_s = -0.4 V~-0.5 V.

Large scale STM image in Figure S1 shows that the "nanowaves" and "nanowires" can coexist on surface as different domains. The "nanowaves" are a guest-host nanoarchitectures where four or two TDPT can nest inside cavities as guest molecules. In some cases, the nano cavities are observed to be empty where no guest molecules are trapped.

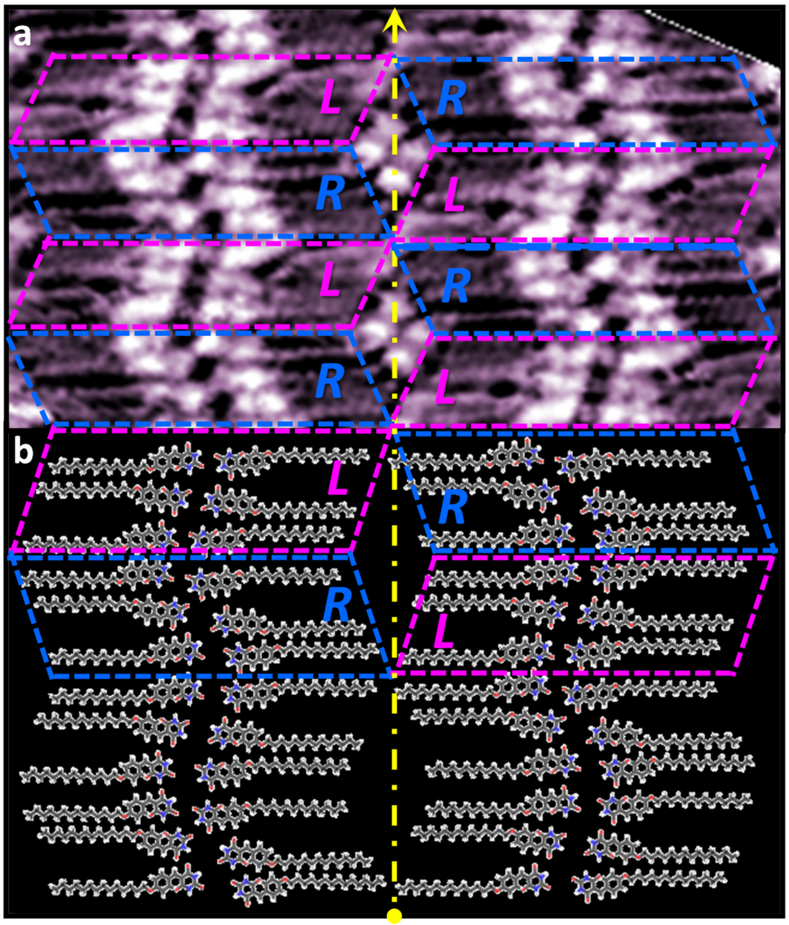

Figure S2. STM images of TDPT "nanowave" and its heterochirality is indicated where the surface plane is considered as the 2D mirror.

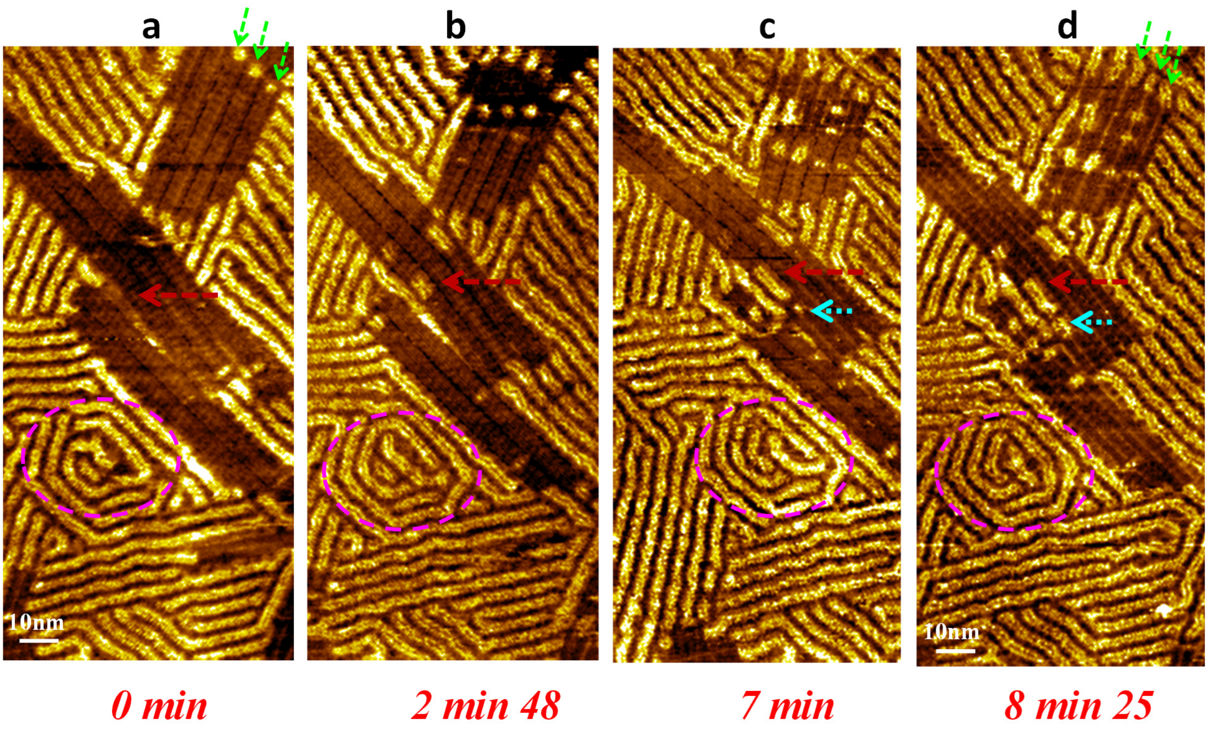

Figure S3. Continuous real-time STM images of TDPT wires deposition on HOPG surface in a 9-min time window. The red, blue and the green arrows indicate where the wires fill the surface cavities. The pink dashed circles reveal the movement of the wires. 150×75 nm^2, I_t = 30 pA, U_s = -0.4V.

Figure S3 a-d are a series of STM images recorded in the time scale of around 9 minutes which reveals molecular deposition at its initial formation stage. We observe that two very short molecular wires start to be absorbed on an empty area (Figure 5b) (no interaction to the neighboring molecules) which reveals that the molecule/substrate interaction is an important driving force of the deposition. Inside the pink circles, the wires change in forms which is probably attributed to the molecules exchange between those on surface and in solution at the solid liquid interface.
